# Serologic Analysis of Returned Travelers with Fever, Sweden

**DOI:** 10.3201/eid1511.091157

**Published:** 2009-11

**Authors:** Helena H. Askling, Birgitta Lesko, Sirkka Vene, Angerd Berndtson, Per Björkman, Jonas Bläckberg, Ulf Bronner, Per Follin, Urban Hellgren, Maria Palmerus, Karl Ekdahl, Anders Tegnell, Johan Struwe

**Affiliations:** Karolinska Institute, Stockholm, Sweden (H.H. Askling, K. Ekdahl); Karolinska University Hospital, Stockholm (H.H. Askling, U. Bronner, U. Hellgren); Swedish National Board of Health and Welfare, Stockholm (B. Lesko, A. Tegnell); Swedish Institute for Infectious Disease Control, Stockholm (B. Lesko, S. Vene, A. Berndtson, J. Struwe); Malmö University Hospital, Malmö, Sweden (P. Björkman); Lund University Hospital, Lund, Sweden (J. Bläckberg); Linköping University Hospital, Linköping, Sweden (P. Follin); County Hospital Ryhov, Jönköping, Sweden (M. Palmerus); European Centre for Disease Prevention and Control, Stockholm (K. Ekdahl); 1 These authors contributed equally to this article.

**Keywords:** Traveler, fever, serology, influenza, rickettsia, dengue, leptospirosis, bacteria, viruses, dispatch

## Abstract

We studied 1,432 febrile travelers from Sweden who had returned from malaria-endemic areas during March 2005–March 2008. In 383 patients, paired serum samples were blindly analyzed for influenza and 7 other agents. For 21% of 115 patients with fever of unknown origin, serologic analysis showed that influenza was the major cause.

Many travelers who return from tropical countries have fever of unknown etiology (*1**–**11*). Earlier studies focusing on fever in returning travelers have used an observation study design with no standardized diagnostics (*1**–**11*). With the exception of studies generated from the GeoSentinel database (*2**,**8*), all are single-center studies. In Sweden, guidelines from the National Board of Health and Welfare advise febrile travelers returning from malaria-endemic areas to be examined at departments of infectious diseases. The objective of this multicenter study was to investigate causes of unknown fever by uniformly analyzing paired serum samples.

## The Study

The study took place in Sweden from March 14, 2005 through March 14, 2008 at 5 hospitals that had infectious diseases departments. Inclusion criteria were travel within the past 2 months to a malaria-endemic area as defined by the World Health Organization, age >18 years, documented temperature >38°C at admission or within the previous 2 days, and a decision by the examining clinician to obtain a blood film for suspected malaria.

Participants were identified either through prospective case finding at emergency rooms and outpatient clinics or through retrospective case finding of eligible patients who had not been included in the prospective case finding; these patients were identified through listings of all performed malaria diagnostics. All included patients had been subject to diagnostic investigations (e.g., cultures, serologic analysis, radiographs) on the basis of clinical symptoms and signs as part of routine procedures at each hospital. An infectious diseases specialist at each study site confirmed the diagnosis based on results of investigations performed. The following variables were recorded for all patients: age, gender, travel history (destination, duration, and purpose), diagnosis, and if applicable, days of hospitalization.

Information about pretravel immunizations and time between return to Sweden and onset of symptoms was available only in the group of prospectively included patients. Travel destinations were grouped as Africa, Asia, and America. Purpose of travel was divided into 3 categories: tourism, Swedish residents originating from a malaria-endemic country and visiting friends and relatives in their country of origin, or other.

Paired serum samples from prospectively included patients were blindly analyzed for antibodies to influenza A and B viruses, dengue virus, chikungunya virus, *Brucella* spp., *Leptospira* spp., *Coxiella burnetii*, *Rickettsia* spp., spotted fever group (SFG) rickettsia, and typhus, respectively. If the travel destination was Asia, *Orientia tsutsugamushi* and Japanese encephalitis virus were also analyzed (Figure). A >4-fold rise in reciprocal antibody titer against a relevant pathogen was considered a positive result. Comparisons between 2 groups were made by using univariate statistics (χ^2^ test); a p value <0.05 was considered significant. The study was approved by the regional Ethics Committee at Karolinska Institute, Stockholm.

**Figure:**
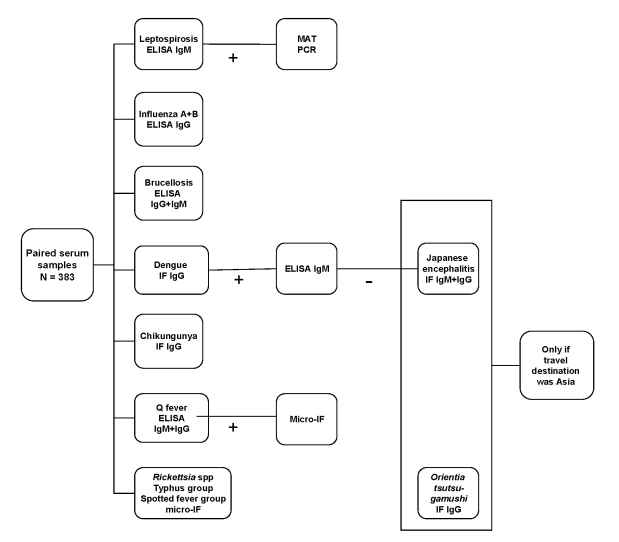
Flow chart of serologic methods performed blindly on all paired serum samples (n = 383), Sweden. Ig, immunoglobulin; MAT, microscopic agglutination test; IF, immunofluorescent.

In 1,432 febrile travelers, the inclusion criteria were fulfilled. A total of 514 patients were identified through prospective case-finding, and 383 of those agreed to be further tested by using blinded serologic analysis; 918 patients were retrospectively identified. Characteristics of these groups are shown in Table 1. Among the entire group (n = 1,432) before results of additional blinded serologic analysis were obtained, unknown fever was diagnosed in 34%, febrile gastroenteritis in 24%, malaria in 6%, influenza in 3%, and dengue fever in 2.5%. In the 383 prospectively included patients, the diagnosis was unknown fever in 115 (30%); additional serologic analysis established a diagnosis in 24 (21%) of these patients.

**Table 1:** Characteristics of 1,432 febrile travelers returning from tropical countries, Sweden, March 2005–March 2008*

Characteristics	Patients with routine investigations		Prospectively identified patients with routine investigation + additional serologic analysis, n = 383
	Prospectively identified, n = 131	Retrospectively identified, n = 918	
Median age, y (range)	32 (18–65)	36 (18–84)	37 (18–76)
Median duration of stay, d	20	21†	20
Female gender	56 (43)	420 (46)	162 (42)
Travel to Africa	69 (53)	430 (47)	199 (52)
Travel to Asia	53 (40)	427 (46)	169 (44)
Travel to America	10 (8)	63 (7)	20 (5)
Tourists	76 (58)	581(63)‡	247 (64)
VFR	10 (8), p = 0.05§	126 (14)‡	20 (5), p<0.0001§
Pretravel influenza immunization	8 (6)	NA	53 (14)
Hospitalized after return to Sweden	37 (28)	258 (28)	123 (32)

*Values are no. (%) patients except as indicated. Some travelers visited >1 region, making the percent sum >100%. VFR, visiting friends and relatives (Swedish residents who were born in a malaria-endemic country and who had visited friends and relatives in their country of origin); NA, not applicable. †In 115 patient files, this information was missing. ‡In 39 patient files, information on type of travel was missing. §Compared with retrospectively identified patients.

The most common diagnosis was influenza (n = 12) followed by SFG rickettsial infection (n = 5), dengue fever (n = 3), leptospirosis (n = 2), Q fever (n = 1), and rickettsial infection caused by *O. tsutsugamushi* (n = 1). A positive serologic result added a co-infection to 23 patients with a diagnosis of illness other than unknown fever; these co-infections were influenza (n = 14), dengue fever (n = 3), typhus group rickettsial infections (n = 2)*,* SFG rickettsial infection (n = 2), leptospirosis (n = 1), and chikungunya fever (n = 1). All infections diagnosed by additional blinded serologic analysis were mild and self-limiting, and the main symptom was fever without typical clinical signs. Fever of unknown etiology was diagnosed in 24% and influenza in 9% of the patients with additional serologic analysis, compared with 35% and 4%, respectively, in the group with routine investigations only (Table 2).

**Table 2:** Final diagnosis of febrile travelers returning from tropical countries, Sweden, March 2005–March 2008*

Final diagnosis	Additional serologic analysis, n = 383, no. (%) patients	Routine investigations only, n = 1,049, no. (%) patients	p value
Fever of unknown etiology	91 (24)	372 (35)	<0.0001
Influenza	34 (9)	38 (4)	<0.001
Dengue fever	17 (4)	27 (3)	NS
Rickettsial infection	17 (4)	15 (1)	<0.001
Leptospirosis	4 (1)	3 (0.2)	NS
Q fever	3 (0.7)	0	0.004
Chikungunya fever	1	0	NS

*NS, not significant.

*Thirty-six patients in the prospectively included group (n = 514) had influenza diagnosed by both routine examination and additional serologic analysis. Eighteen of the 36 became ill with fever either just before returning to Sweden or within 1 day of arrival, indicating that they acquired the infection abroad*; *5 had been home 1–2 days, indicating that the infection could have been acquired either during travel or after the return; and 13 patients had returned from travel >3 days before falling ill with fever, indicating that they most likely became infected in Sweden.* Twenty-five of the 36 influenza patients had verified influenza A infection, and 11 had influenza B infection. Nine (25%) patients became ill after returning from a trip occurring well outside the influenza season of the northern hemisphere; 7 had visited Africa, and 2 had traveled to Asia.

## Conclusions

Influenza is often missed in routine diagnostics of febrile travelers. Our findings highlight the role of travel in the global spread of influenza and corroborate the findings of influenza in travelers by others (*12**,**13*). Apart from influenza, the most common diseases missed in routine investigations were rickettsial infections, dengue fever, and leptospirosis. Our study adds a new approach by using a systematic collection of paired sera. The retrospective case finding is not fully comparable with the prospective inclusion of patients, and we are missing some retrospective data on type and length of travel. These missing data are, to some extent, compensated by a careful retrospective review of all 918 patients’ files, the finding that the characteristics of the 2 groups are similar, and the similarity of the routine investigations for both groups.

Additional blinded serologic analyses were performed by using the same method in the same laboratories. The proportion of final diagnoses with fever of unknown etiology was high compared with that of other studies, even after results of the additional serologic analysis (*1**–**8**,**11*). This large proportion of fever with unknown etiology may be explained by the unselected study population in a hospital setting and by a high patient turnover; febrile travelers with a negative malaria film and in good clinical condition are often sent home without extensive investigations or follow up.

To estimate the number of nasopharyngeal swabs taken as a routine test, we retrospectively reviewed a sample of 217 patient files and found that 31 (14%) had been tested for influenza; 6 of those tests yielded positive results. Age, gender ratio, destinations, duration of travel, and hospitalization rates were similar to those of recent studies (*3**,**7**,**8*). The finding of undiagnosed rickettsial infections shows that symptoms are often nonspecific, and serologic response often delayed (*14*).

Our results indicate that leptospirosis is an underestimated cause of fever in returned travelers and is not only related to extreme sports (*15*). The relatively low frequency of additional rickettsial infections, dengue, and leptospirosis indicates that paired sera should not be routinely recommended without a specific clinical suspicion. However, this study supports the theory that diseases with classic clinical findings according to text books can also manifest as fever only. Influenza should always, in all seasons, be considered when diagnosing illness in returning febrile travelers.
